# Assessing Patient Acceptance of Being Offered a Virtual Calm Room in Psychiatric Inpatient Care: Mixed Methods Study

**DOI:** 10.2196/75961

**Published:** 2025-12-29

**Authors:** Maria Ilioudi, Steinn Steingrimsson, Sara Wallström, Almira Osmanovic Thunström, Philip Lindner, Michael Ioannou, Örjan Falk, Lilas Ali

**Affiliations:** 1 Institute of Health and Care Sciences, Sahlgrenska Academy, University of Gothenburg, Arvid Wallgrensbacke 1, Gothenburg, 405 30, Sweden, 1 031-786 00 00; 2 Department of Psychiatry for Affective Disorders, Region Västra Götaland, Sahlgrenska University Hospital, Gothenburg, Sweden; 3 Institute of Neuroscience and Physiology, Sahlgrenska Academy, University of Gothenburg, Gothenburg, Sweden; 4 Centre for Person-Centred Care (GPCC), University of Gothenburg, Gothenburg, Sweden; 5 Department of Forensic Psychiatry, Region Västra Götaland, Sahlgrenska University Hospital, Gothenburg, Sweden; 6 Centre for Ethics, Law and Mental Health (CELAM), University of Gothenburg, Gothenburg, Sweden; 7 Institute of Neuroscience and Physiology, Sahlgrenska Academy, University of Gothenburg, Gothenburg, Sweden; 8 Centre for Psychiatry Research, Department of Clinical Neuroscience, Karolinska Institutet & Stockholm Health Care Services, Stockholm, Sweden

**Keywords:** acceptance, inpatient, psychiatric care, virtual reality, VR

## Abstract

**Background:**

Implementing new technologies in health care settings is often a complex and challenging process. Virtual reality (VR) has demonstrated promising results in terms of feasibility, acceptability, and effectiveness across various health conditions. VR can provide calming environments to enhance patients’ well-being in psychiatric inpatient care. However, little research has been done on patients’ acceptance of VR technology in psychiatric care in the real-world setting.

**Objective:**

This study aimed to explore patients’ experiences of being offered the use of a virtual calm room when feeling anxious or worried in a psychiatric inpatient setting.

**Methods:**

A mixed methods design was used, with a qualitative to quantitative approach. The data were gathered through individual interviews (n=10) and a 3-item rating scale ranging from 1 to 5 (n=59). The qualitative findings were then validated within a larger population using the quantitative data.

**Results:**

The majority of participants reported being satisfied with the option of using VR. Their initial impressions of the virtual calm room were that it seemed like a creative and stimulating environment that could potentially have a positive impact on them. They expected the VR experience to enhance their feelings of relaxation and concentration. The participants highlighted human interaction as a particularly valuable aspect to consider when implementing VR, emphasizing its role in enhancing the overall experience and ensuring a sense of connection and support throughout the process. Participants were generally positive in the answers to the survey with a high score of feeling VR to be a modern method to manage anxiety (mean 4.3, SD 0.98), low levels of experiencing difficulty (mean 2.1, SD 1.4), and would generally consider future use (mean 4.4, SD 1.1).

**Conclusions:**

The qualitative findings highlighted patients’ openness to innovative methods for enhancing their engagement in the psychiatric inpatient setting. Patients expressed a desire for increased availability of the virtual calm room. However, maintaining a balance between innovative technologies and human support is crucial for the successful implementation of such methods. Quantitative results demonstrated high acceptance of the option of using the virtual calm room, with no significant difficulties reported.

## Introduction

Implementing new technologies in health care settings is often challenging, but there is much to gain from doing so. Virtual reality (VR) is one such technology. Despite decades of research support for its clinical application, it has only recently begun to be incorporated into real-world health care [1]. VR, now considered a subtype under the umbrella term “Extended Reality,” refers to headset technology capable of generating a sense of being present in a virtual world. A recent clinical application of VR provides calming virtual environments that can be offered in psychiatric inpatient settings to enhance patients’ sense of safety. The application fosters a recovery-focused atmosphere that is the virtual equivalent of physical calm rooms [2]. Both physical and VR calm rooms offer nontraditional therapeutic interventions that improve patients’ overall admission experience and well-being [3,4].

Research has already demonstrated the feasibility, acceptability, and effectiveness of clinical VR in regular healthcare settings [5]. It has also been shown to be a promising option in both inpatient [4,6,7] and outpatient [8] psychiatric care settings. It is increasingly being used in patients with conditions such as phobias [9], anxiety [10], post-traumatic stress [11], depression [12], delusions [13], and anorexia [14]. VR applications have been specifically designed to induce a sense of relaxation [15], lower anxiety, and increase well-being and independence [16,17]. Where safety concerns sometimes preclude the construction of physical calm rooms in psychiatric healthcare settings, VR technology could provide an alternative option [1].

Health care services are under near-constant pressure to increase both the quality and cost efficiency of care [18]. However, research has also uncovered significant challenges in implementing new technologies on a large scale [19]. Given that the health care system offers services co-created by health care professionals and people seeking help, improving health care services requires considering innovative ways to involve both staff and patients, along with new organizational forms, structures, and community engagement [20]. Patient and public involvement is also increasingly being recognized as beneficial in health care research. Participatory research design highlights users’ experiences to create new systems based on their collective interpretations of research, thereby enhancing its usability. Users’ knowledge is highly valued, grounded in a strong ethical framework, and can be leveraged to design new tools [21].

VR technology has, in theory, made it feasible to implement calm rooms in routine psychiatric care without the need for a dedicated room, trained and available staff, and other practical obstacles associated with the traditional alternative [4]. To maximize the probability of long-term, sustainable, and successful outcomes, this implementation should arguably be performed in a bottom-up manner, involving the intended users from the start to ensure that the technology at hand addresses actual problems and adds value [22]. Including the end user’s perspective and therapists’ feedback throughout the design, development, and real-world implementation of VR applications will likely result in enriched applications with elements familiar to and preferred by end users [23].

VR applications can offer individualized experiences for patients with various mental disorders [24] and exposure to relaxing VR scenes may provide an effective relaxation method to support and enhance psychiatric treatments [25,26]. In previous studies, patients reported high satisfaction with and acceptance of VR technology [8,27,28]. However, few studies have evaluated patients’ acceptance of being offered the option of VR technology in psychiatric inpatient settings. A previous qualitative study conducted by the research team showed that use of a virtual calm room made a positive impression on patients. However, the earlier study did not explore the users’ experiences of being offered such an option instead of traditional pharmacological treatment [13]. Therefore, the aim of this study was to explore patients’ views on the option of being offered the use of a virtual calm room when feeling anxious or worried in a psychiatric inpatient setting.

## Methods

### Study Design

This study is part of a larger research project aimed at exploring the effect of VR and physical calm rooms in psychiatric inpatient care. The larger project also includes a quasi-randomized controlled trial and a qualitative interview study (trial registration NCT03918954) [4,7]. The participants included in this study were not part of the samples in any of the previous studies. This study adopted a mixed methods design using the qualitative to quantitative approach, with qualitative data being used to interpret the quantitative data [29].

Mixed methods research involves the integration of qualitative and quantitative data [29,30]. This methodology is appropriate when neither qualitative nor quantitative data adequately address a complex research question, when a phenomenon needs to be explored in depth, when different types of cases need to be explored and compared, as well as when a program needs to be developed, implemented, and evaluated. Findings from one approach are enhanced by the other approach [29,30].

In this study, qualitative data about individual patients’ experience of being offered the use of the VR calm room were collected through interviews. In the second step, data were gathered from statements included in an evaluation form. In the last step, all the collected data were integrated into the interpretive process.

### Sample

Patients who had already been admitted to psychiatric inpatient care were asked if they were interested in using a VR or physical calm room and willing to provide ratings as well as participate in interviews. The inclusion criteria were individuals aged 18 years or older and having been admitted to the inpatient psychiatric care ward specializing in bipolar disorder at Sahlgrenska University Hospital in western Sweden. No exclusion criteria were predefined. A total of 59 participants received a 3-item evaluation form, including 10 participants who were willing to be interviewed and were subsequently recruited. Data were collected between 2022 and 2023.

### Patient and Public Involvement

This study incorporates a participatory design that assumes that patients and health care professionals are involved in the study design and development stage [21]. Extensive iterative consultations with the users took place during the development and design of the VR calm room. At this stage, users gave feedback on the application components and shared ideas to make the development more user-friendly and suitable for the clinical setting.

### Procedure

**Figure 1.**
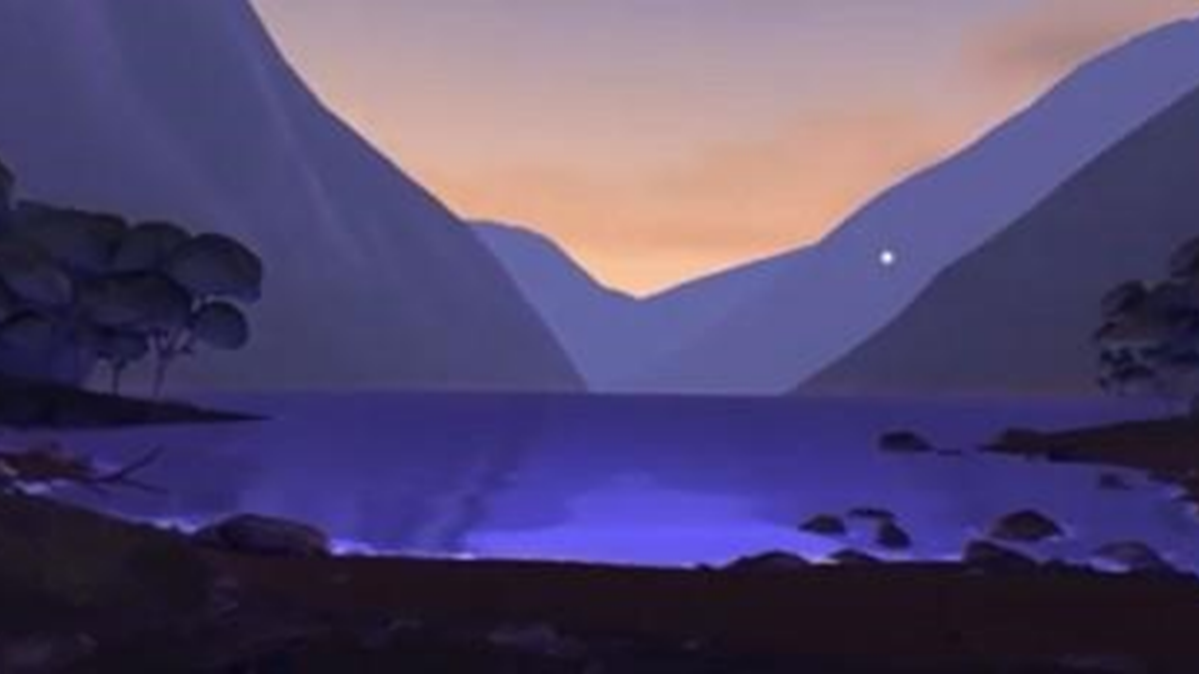
Example of a calming scene.

Patients who had been admitted to a psychiatric care setting specializing in bipolar disorder used the virtual calm room. Health care professionals supported the patients with instructions on the VR headset’s functions and how to navigate in the virtual environment. To engage with the device, patients used a wireless, 3 degrees-of-freedom VR head-mounted display (Oculus Go; Meta Reality Labs) running the Calm Place app developed by Mimerse. The application consists of relaxing nature environments such as a beach, a forest, and a desert (Figure 1). The application offers users different options to engage with, including breathing exercises, mindfulness programs, and relaxing music. They can also choose to just observe relaxing images of natural environments. Natural sounds (eg, birds, rain, and ocean waves) can be added according to the user’s preference.

### Data Collection

#### Qualitative Data

After the patients used the VR calm room, the first author (MI) conducted individual interviews with 10 patients. Each interview lasted between 7 and 19 minutes. None of the participants expressed any concerns or distress either during the interview or afterward. Participants were encouraged to be as specific as possible about their experience of being offered the use of the VR calm room. Interviews were conducted using an interview guide, which aimed to gather data on patients’ reflections and reasoning. Participants were asked questions about their expectations when offered the VR calm room, whether they found it helpful, whether they experienced it as a modern method, how they experienced the health care professionals’ attitudes to implementing the VR calm room, and whether they would be interested in using this method in the future.

#### Quantitative Data

Patient characteristics and demographics were self-reported and collected directly from the participants. To evaluate the acceptance of the virtual calm room, 59 patients, including the 10 participants who were interviewed, responded to 3 statements in a standardized evaluation form: “*this feels like a modern strategy to reduce anxiety*,” “*I found it difficult to access the virtual calm room*,” and “*If presented with the choice again, I would consent to using it*.” Responses were recorded using a 5-point Likert scale ranging from 1 (“do not agree”) to 5 (“fully agree”). The statements were carefully formulated to capture key components of intervention acceptance, including affective attitude, ethical considerations, perceived burden, intention to use, intervention coherence, opportunity costs, perceived effectiveness, and self-efficacy. This approach, which had been formulated for a previous pragmatic randomized controlled trial [31], ensured an evaluation of patients’ experiences and attitudes toward the virtual calm room [32].

#### Data Analysis and Synthesis

Data from the interviews were analyzed using qualitative inductive content analysis. Qualitative content analysis involves breaking down data into smaller units for in-depth examination [30]. The primary challenge in qualitative research is determining whether to focus the analysis on manifest or latent content, as both approaches involve interpreting the text. Another aspect of interpretation is that although researchers are influenced by their personal histories, they must allow “the text to speak for itself” [29]. A key characteristic of this method is its emphasis on the context and the subject matter. The content analysis followed the steps outlined by Graneheim and Lundman [33], which include selecting the unit of analysis, identifying meaning units, condensing and abstracting data, defining content areas, and developing codes, categories, and themes. We employed a descriptive qualitative approach to generate findings that closely align with the data. The transcribed text was read multiple times by the first author to gain an understanding of its content. The research group then discussed the content collaboratively to identify meaning units within the text. In the next step, the data were condensed and abstracted by MI and LA to create codes. Subthemes with similar content were developed and subsequently grouped into larger themes. The analysis was a dynamic process, with authors revisiting each step as needed. Finally, the authors discussed the overall organization of meaning units, codes, and subthemes, reaching a consensus on the main themes.

Data on background characteristics as well as the evaluation forms were analyzed using descriptive statistics. Both qualitative and statistical analyses were performed on the collected data. The results from the qualitative and quantitative analyses are presented separately. Patterns and relationships between the qualitative and quantitative data were identified, and details from the 2 methods were integrated to provide a comprehensive understanding of the findings. Following this, data from both sources were synthesized to draw broader conclusions. All authors were actively involved in the analysis process, collaboratively discussing and interpreting the various outcomes to ensure the validity and depth of the findings.

### Ethical Considerations

Ethical approval for the study was granted by the Swedish Ethical Review Authority (318‐18 and amendment 2023-06878-02). Written informed consent was obtained from all participants, who could choose to end their use of the calm room at any time without needing to provide a reason. If a participant had previously exhibited any risk of self-harm or if there was any suspicion that their use of the calm room could be harmful, they were not granted access. Participants admitted to a psychiatric inpatient ward may receive compulsory psychiatric care. The research group was prepared to intervene if any participant was negatively affected by the questions asked or by their participation in the study in any way. All data were treated confidentially.

## Results

### Demographic and Clinical Characteristics

The demographic and clinical characteristics of the participants are summarized in Table 1. The sample consisted of 59 participants, of whom 17 (29%) were male participants and 38 (64%) female participants (n=4, 7% participants did not specify their sex). Participants’ ages ranged from 18 to 74 years. The majority have been diagnosed with bipolar disorder.

**Table 1.** Demographic and clinical characteristics of survey participants (N=59^a^).

Characteristic	Value
Age (y), mean (SD; range)	42.9 (14.3; 18‐74)
Gender^b^, n (%)	
Man	17 (29)
Woman	38 (64)
Left blank	4 (7)
Self-reported psychiatric disorder^c^, n (%)	
Bipolar disorder^b^	34 (58)
Depression^c^, n (%)	7 (12)
Other	3 (5)
Left blank	14 (24)

a Interviewees are also included in the population.

b Three participants also reported emotionally unstable personality disorder, with one of them also having attention-deficit/hyperactivity disorder.

c Three participants also reported anxiety disorders.

### Results From Qualitative Interviews

The analysis of the qualitative data resulted in the identification of 2 themes dealing with the overall experience of being offered a technical solution when experiencing anxiety or worry while in the inpatient ward: *innovative thinking in providing other options* and *the value of human interaction*.

#### Innovative Thinking in Providing Other Options

The participants expressed feelings of being trapped in the psychiatric inpatient ward and wanting creative ways to stimulate their minds. Their first thought when offered use of the VR calm room was that it seemed like a creative environment that might offer some positive stimulation. They anticipated that the VR technology might enhance their ability to relax and concentrate. Patients described the virtual calm room as a solution to their need for stimulation when they could not leave the ward. It provided an opportunity for a moment of relaxation, a brief time when they could experience harmony and well-being.

*I have gained a new experience that I never thought I would have . . . I feel like I have so many creative ideas, and I would really love to work with VR in some other way*.

[P1]

All the participants, both those who had used VR before and those using it for the first time, were satisfied with being offered access to the VR calm room. They perceived the room as easily accessible, and their first thought was that they were curious and open to experiencing a new method. Even participants who had never tried VR technology before did not report any hesitation about trying the VR calm room. Some participants stated that it was better than they had expected and that they would recommend it to other patients as an alternative option for relieving anxiety. The participants also found the process uncomplicated, with the lack of obstacles allowing them independence in using the device.

Some participants indicated that they wanted the VR session to become an everyday activity because they found it an attractive way to get through their challenging inpatient stay. It was an alternative that could help them focus on the present. The participants also said that the device seemed to be a good and valuable technical alternative to medication. Some participants mentioned other alternatives they had tried such as painting or taking walks in the yard. The VR experience was considered a new and interesting way to spend their time on the psychiatric inpatient ward.

#### The Value of Human Interaction

The participants appreciated being offered new technology; however, they were also clear that they preferred less medical support and more human support. They considered human contact with health care professionals to be an important part of their care. They valued their time with their contact person in the inpatient ward and emphasized that continued communication was important for their recovery. The patients also wanted the contact person to introduce them to the ward’s routines and new alternatives such as the VR calm room.

*I like alternative medicine; I appreciate things that have no side effects or fewer side effects. And then there’s the idea that if you learn something in the virtual world, maybe you can apply that technique outside the virtual world on your own after practicing it several times. So, I thought it was very positive*.

[P6]

The participants emphasized that they appreciated being listened to by the professionals on the inpatient ward, as well as being offered VR support and being shown how to use the self-guided applications. For some, the included mindfulness program distracted them from their anxiety. The participants also mentioned wanting more methods to increase body awareness, breathing techniques, or anxiety management to be available for them on the ward.

### Ratings

Table 2 shows the results of the survey. The findings reveal that most participants did not encounter any difficulties using the VR technology, reporting a median score of 1 (on a scale where lower values indicate fewer difficulties). In addition, participants viewed VR technology as a modern treatment method for mental disorders, with a median score of 5 indicating a positive view. Most participants would choose to participate in the intervention again, if given the opportunity.

**Table 2.** Responses to an evaluation of acceptance of virtual calm room to 3 questions on a 5-point agree–disagree Likert scale (1=do not agree at all to 5=fully agree).

Question	Responders	Median	mean (SD)	Likert scale (%)				
				1	2	3	4	5
This feels like a modern strategy to reduce anxiety	59	5	4.3 (0.98)	3	2	12	29	54
I found it difficult to access the virtual calm room	58	1	2.1 (1.4)	52	17	16	5	10
If I could choose again, I would consent to using the virtual calm room	58	5	4.4 (1.1)	4	0	4	9	41

## Discussion

### Principal Findings

This mixed methods study explored patients’ experiences of being offered the use of a virtual calm room in a psychiatric inpatient setting. The qualitative findings showed that patients expressed a need for creative stimulation and engagement. They appreciated the innovative use of VR technology, noting that it helped alleviate their anxiety and enhanced their relaxation and concentration.

Quantitative data further supported these findings in a larger population, showing that the virtual calm room was perceived as a modern treatment option for mental disorders, with a median score of 1 indicating minimal difficulties in usage. These results align with previous studies that have found that participants expressed a desire for new treatment options, such as VR technology, that go beyond traditional medication [7], and that they found virtual relaxation helpful, enjoyable, and relaxing [34-36]. Previous research shows that both health care providers and patients regarded VR technology as having the potential to bring something new and unique to psychiatric care [37].

The quantitative data show that most participants did not experience any difficulties using the virtual calm room. They felt a sense of autonomy while using the device and found it easy to initiate and follow the session. These findings align with an earlier study that reported that patients demonstrated high acceptance of VR technology and perceived it as uncomplicated to use [38].

Despite the potential and expanding use of VR technology, its use in clinical psychiatric care is limited, as is research on data with deeper insights [1]. This may be because implementing technology-based innovations can be challenging due to clinical risks and safety concerns [39]. While professional concerns about the implementation of VR technology are no longer the major barrier to its use in clinical contexts [40], evidence-based clinical guidelines and training are needed to ensure its effective integration into psychiatric care [39,41].

A positive patient experience, a sense of safety, and a strong professional relationship have been shown to be key elements of VR treatment [42]. In an earlier study, clinicians mentioned the need to add VR tasks that target patients’ emotional states and to gather evidence before implementing the treatment. Patients felt that VR treatment should be adapted to their preferences and made more accessible and convenient in terms of how, when, and where it is used [43].

Patients expressed a willingness to use the virtual calm room again and indicated a desire for new and engaging ways to occupy their time on the ward. While the VR relaxation experience was perceived as more effective than conventional relaxation exercises for immediately alleviating negative affective states [26,44], this finding raises important questions about the sustainability and accessibility of such technology in psychiatric settings. Although the immediate benefits of VR are promising, reliance on technological interventions may inadvertently overshadow traditional therapeutic methods that are also vital for patient recovery. In previous research, treatments based on VR technology have not clearly shown superiority over conventional treatments [24]. Previous studies have also indicated that patients have maintained their ongoing pharmacological treatment alongside VR treatment, thus it is difficult to evaluate on its own [45]. Furthermore, not all patients may feel comfortable or able to engage with VR technology, potentially leading to disparities in treatment experiences. Therefore, while integrating VR into psychiatric treatment has the potential to enhance patient care, it is crucial to consider how to balance innovative methods with the diverse needs and preferences of all patients, ensuring that no one is left behind in the pursuit of improved mental health outcomes.

### Strength and Limitations

This mixed methods (qualitative to quantitative) study has several limitations. The qualitative data came from a small sample of only 10 participants because of the inpatient psychiatric context, and the study aim did not require extensive, in-depth descriptions. However, the qualitative data complemented the quantitative data, enabling the research to effectively address its objective. The quantitative part of the study relied on an evaluation form that had not been validated as it was adapted from a previous clinical study. Furthermore, the evaluation form included a limited number of statements to maximize the sample size. Demographic data for the qualitative and quantitative parts were presented together, since participants self-reported their characteristics. The study was conducted within an affective disorder department, representing a limitation regarding the generalizability of the results to a wider psychiatric population. Despite these limitations, the mixed methods approach proved to be a strength of the study: by integrating qualitative insights with quantitative findings, the study provided a comprehensive understanding of patients’ perspectives on being offered the use of a virtual calm room in a psychiatric inpatient setting. This methodology offered valuable insights into patient acceptance and the potential benefits of this intervention.

### Conclusions

The qualitative results revealed patients’ openness to VR calm rooms to improve their engagement with psychiatric inpatient care. The quantitative results demonstrated patients’ high acceptance of virtual calm rooms and did not indicate any noteworthy difficulties. Patients wanted increased availability of virtual calm rooms. However, a balance between innovative methods and human support is crucial for the implementation of technologies. Further research based on codesign may provide patients with opportunities to adapt their preferences and develop more convenient innovative solutions.

## Abbreviations

VR: virtual reality
